# Could an Unrelated Live Attenuated Vaccine Serve as a Preventive Measure To Dampen Septic Inflammation Associated with COVID-19 Infection?

**DOI:** 10.1128/mBio.00907-20

**Published:** 2020-06-19

**Authors:** Paul L. Fidel, Mairi C. Noverr

**Affiliations:** a Department of Oral and Craniofacial Biology, Louisiana State University Health—School of Dentistry, New Orleans, Louisiana, USA; b Department of Microbiology and Immunology, Tulane University School of Medicine, New Orleans, Louisiana, USA; Harvard Medical School

**Keywords:** MMR, clinical trial, inflammation, COVID-19, live attenuated vaccines, myeloid-derived suppressor cells, sepsis, trained innate immunity

## Abstract

We propose the concept that administration of an unrelated live attenuated vaccine, such as MMR (measles, mumps, rubella), could serve as a preventive measure against the worst sequelae of coronavirus disease 2019 (COVID-19). There is mounting evidence that live attenuated vaccines provide nonspecific protection against lethal infections unrelated to the target pathogen of the vaccine by inducing “trained” nonspecific innate immune cells for improved host responses against subsequent infections. Mortality in COVID-19 cases is strongly associated with progressive lung inflammation and eventual sepsis. Vaccination with MMR in immunocompetent individuals has no contraindications and may be especially effective for health care workers who can easily be exposed to COVID-19. Following the lead of other countries conducting clinical trials with the live attenuated Mycobacterium bovis BCG (BCG) vaccine under a similar concept, a clinical trial with MMR in high-risk populations may provide a “low-risk–high-reward” preventive measure in saving lives during this unprecedented COVID-19 pandemic.

## Abstract (precis)

We propose the concept that administration of an unrelated live attenuated vaccine, such as MMR (measles, mumps, rubella), could serve as a preventive measure against the worst sequelae of coronavirus disease 2019 (COVID-19). There is mounting evidence that live attenuated vaccines provide nonspecific protection against lethal infections unrelated to the target pathogen of the vaccine by inducing “trained” nonspecific innate immune cells for improved host responses against subsequent infections.

## OPINION/HYPOTHESIS

**FIG 1:**
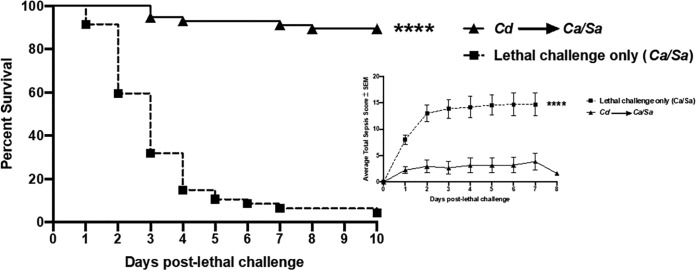
Protection against lethal polymicrobial-community-mediated sepsis by vaccination with an avirulent yeast strain. Mice were vaccinated intraperitoneally with avirulent (live attenuated) Candida dubliniensis (*Cd*) followed by a lethal challenge with Candida albicans *and* Staphylococcus aureus (*Ca*/*Sa*) intraperitoneally 14 days later. Survival data are shown for 8 independent experiments performed with groups of 10 mice each. The inset shows sepsis scoring during the observation period after lethal challenge. Animals were monitored daily using a modified mouse clinical assessment score for sepsis (M-CASS) scoring system that included fur aspect, posture, activity, and chest and eyelid movement. Animals with a combined daily score of 8+ were humanely euthanized. Data were statistically analyzed using the Mantel-Cox log rank test (survival curves) and two-way analysis of variance (ANOVA) with Bonferroni *post hoc* tests (sepsis scoring) (****, *P* < 0.0001). SEM, standard errors of the means.

There is mounting evidence that the use of live attenuated vaccines commonly administered during childhood also provides beneficial nonspecific effects (NSE), including reduced mortality and hospitalization due to unrelated infections (1, 2). In this Commentary, we outline a rationale to support the use of live attenuated vaccines, such as MMR (measles, mumps, rubella), as a preventive measure against the pathological inflammation and sepsis associated with coronavirus disease 2019 (COVID-19) infection (3). We emphasize this is strictly a preventive measure against the worst inflammatory sequelae of COVID-19 for those exposed/infected and does not represent an antiviral therapy or vaccine against COVID-19 in any manner. It has been proposed that live attenuated vaccines induce nonspecific effects representing “trained innate immunity” by “training” leukocyte precursors in the bone marrow to function more effectively against broader infectious insults (4). In support of this, work from our laboratory demonstrated that vaccination with a live attenuated fungal strain induces trained innate protection against lethal polymicrobial sepsis (Fig. 1) (5–7). The protection is mediated by long-lived myeloid-derived suppressor cells (MDSCs) previously reported to inhibit septic inflammation and mortality in several experimental models (reviewed in reference 7).

The general concept that the trained innate immunity induced by live attenuated vaccines can limit pathological inflammation is novel but not without precedent. At least 6 clinical trials have been initiated in Europe, Australia, and the United States to test vaccination with Mycobacterium bovis BCG (live attenuated tuberculosis [TB] vaccine) or placebo in high-risk health care workers to determine whether beneficial trained innate responses against COVID-19 can be elicited (https://www.sciencemag.org/news/2020/03/can-century-old-tb-vaccine-steel-immune-system-against-new-coronavirus; https://www.nytimes.com/2020/05/01/opinion/sunday/coronavirus-vaccine-innate-immunity.html). In these trials, the proposed trained innate response is one of immune enhancement that could possibly reduce viral levels and/or sequelae associated with COVID-19, similar to what has been reported for other viral infections (2). In contrast, we propose that the trained innate response includes induction of the MDSCs that can inhibit/reduce the severe lung inflammation/sepsis associated with COVID-19. In either regard, on the basis of data from prior BCG trials in infants, the vaccine-induced trained innate cells remain in the circulation for roughly 1 year (8). Hence, if these innate responses are indeed induced in the current clinical trials, recipients should benefit throughout the acute crisis period of the current COVID-19 pandemic until a conventional vaccine is available or antiviral therapies become more accessible. One caveat concerning BCG vaccination is seroconversion, which is the basis for the TB diagnostic test currently used in the United States. Hence, BCG vaccination is not conducted in the United States. We therefore are proposing the use of the live attenuated MMR vaccine, which has also been found to be associated with beneficial NSE in human populations.

According to the Centers for Disease Control (CDC), there are few contraindications against administration to adults of a live attenuated vaccine such as MMR if the recipient is immunocompetent and not pregnant and has not shown previous allergic responses to vaccination (https://www.cdc.gov/vaccines/vpd/mmr/hcp/recommendations.html). In fact, MMR vaccination is recommended in high-risk adults (i.e., health care workers) and people born before 1957 who did not receive the vaccine as a child. Adults who had received the MMR vaccine in childhood likely still possess antibody titers against the targeted viruses but not the shorter-lived trained innate leukocytes. Hence, at the very least, the MMR vaccine would provide added protection against measles, mumps, and rubella for older adults. But with the added induction of the trained innate cells, the MMR vaccination could provide protection against the worst sequelae of COVID-19. In direct support of this concept, it was recently reported that the milder symptoms seen in the 955 sailors on the U.S.S. *Roosevelt* who tested positive for COVID-19 (only one hospitalization) may have been a consequence of the fact that MMR vaccinations are given to all U.S. Navy recruits (https://www.globenewswire.com/news-release/2020/05/01/2026166/0/en/MMR-Vaccine-May-Reduce-COVID-19-Hospitalization-Rate-According-to-World-Organization.html). In addition, epidemiological data suggest a correlation between subjects in geographical locations who routinely receive live attenuated measles-rubella vaccines such as the commonly available MMR vaccine, and reduced COVID-19 death rates (https://www.researchgate.net/publication/341354165_MMR_Vaccine_Appears_to_Confer_Strong_Protection_from_COVID-19_Few_Deaths_from_SARS-CoV-2_in_Highly_Vaccinated_Populations).

Looking historically at other viral respiratory epidemics and pandemics of seasonal influenza, severe acute respiratory syndrome (SARS), and Middle East respiratory syndrome (MERS), one of the more interesting observations is the drastic difference in mortality rates between children and adults (9). Children are highly susceptible to flu; the CDC estimates that since 2010, the number of flu-related hospitalizations among children younger than 5 years of age has ranged from 7,000 to 26,000 in the United States, with approximately 600 deaths in the past 5 years (10). However, very few children were affected during the SARS (2003) or MERS (2012) coronavirus outbreaks (9) and now as well during the COVID-19 outbreak (https://www.cdc.gov/coronavirus/2019-ncov/specific-groups/children-faq.html). This is likely due to differences in progression, pathogenesis, and cause of mortality between influenza virus and coronavirus infections. With seasonal flu, mortality is in large part the result of secondary infections, including those by bacterial pneumonia, and of exacerbations of underlying chronic conditions (10). With SARS and MERS, mortality is the result of severe pulmonary inflammation and sepsis induced by the virus resulting in eventual organ failure (3). We hypothesize that one reason that children are protected against viral infections that induce sepsis is their more recent and more frequent exposures to live attenuated vaccines (MMR, rotovirus, smallpox, chickenpox, BCG) that can also induce the trained suppressive MDSCs that limit inflammation and sepsis.

As a response to this hypothesis along with the supportive rationale and considerable observational data supporting the hypothesis, we have proposed a randomized clinical trial to be performed with MMR in New Orleans for high-risk health care workers and first responders. In addition, we have been awarded a Fast Grant (as part of Emergent Ventures at the Mercatus Center, George Mason University) to investigate the level of efficacy of MMR versus that of BCG in a nonhuman primate model of COVID-19 infection. If our hypothesis is correct, MMR (booster) vaccination in adults represents a “low-risk–high-reward” preventive measure to save lives during a critical period of the COVID-19 pandemic.

## References

[1] Aaby P, Benn CS 2019 Developing the concept of beneficial non-specific effect of live vaccines with epidemiological studies. Clin Microbiol Infect 25:1459–1467. doi:10.1016/j.cmi.2019.08.011. 31449870

[2] Moorlag S, Arts RJW, van Crevel R, Netea MG 2019 Non-specific effects of BCG vaccine on viral infections. Clin Microbiol Infect 25:1473–1478. doi:10.1016/j.cmi.2019.04.020. 31055165

[3] Zhou F, Yu T, Du R, Fan G, Liu Y, Liu Z, Xiang J, Wang Y, Song B, Gu X, Guan L, Wei Y, Li H, Wu X, J Xu, Tu S, Zhang Y, Chen H, Cao B 2020 Clinical course and risk factors for mortality of adult inpatients with COVID-19 in Wuhan, China: a retrospective cohort study. Lancet 395 doi:10.1016/S0140-6736(20)30566-3. PMC7270627 32171076

[4] Kaufmann E, Sanz J, Dunn JL, Khan N, Mendonça LE, Pacis A, Tzelepis F, Pernet E, Dumaine A, Grenier J-C, Mailhot-Léonard F, Ahmed E, Belle J, Besla R, Mazer B, King IL, Nijnik A, Robbins CS, Barreiro LB, Divangahi M 2018 BCG educates hematopoietic stem cells to generate protective innate immunity against tuberculosis. Cell 172:176–190.e19. doi:10.1016/j.cell.2017.12.031. 29328912

[5] Lilly EA, Ikeh M, Nash EE, Fidel PL Jr, Noverr MC 2018 Immune protection against lethal fungal-bacterial intra-abdominal infections. mBio 9:e01472-17. doi:10.1128/mBio.01472-17. 29339423 PMC5770546

[6] Lilly EA, Yano J, Esher SK, Hardie E, Fidel PL Jr, Noverr MC 2019 Spectrum of trained innate immunity induced by low-virulence Candida species against lethal polymicrobial intra-abdominal infection. Infect Immun 87 doi:10.1128/IAI.00348-19. PMC6652762 31085710

[7] Esher SK, Fidel PL Jr, Noverr MC 2019 Candida/staphylococcal polymicrobial intra-abdominal infection: pathogenesis and perspectives for a novel form of trained innate immunity. J Fungi (Basel) 5 doi:10.3390/jof5020037. PMC6617080 31075836

[8] Biering-Sørensen S, Aaby P, Lund N, Monteiro I, Jensen KJ, Eriksen HB, Schaltz-Buchholzer F, Anne Sofie Pinstrup Jørgensen AS, Rodrigues A, Fisker AB, Stabell Benn C 2017 Early BCG-Denmark and neonatal mortality among infants weighing <2500 g: a randomized controlled trial. Clin Infect Dis 65:1183–1190. doi:10.1093/cid/cix525. 29579158 PMC5849087

[9] Liu W, Zhang Q, Chen J, Xiang R, Song H, Shu S, Chen L, Liang L, Zhou J, You L, Wu P, Zhang B, Lu Y, Xia L, Huang L, Yang Y, Liu F, Semple MG, Cowling BJ, Lan K, Sun Z, Yu H, Liu Y 2020 Detection of Covid-19 in children in early January 2020 in Wuhan, China. N Engl J Med 382:1370–1371. doi:10.1056/NEJMc2003717. 32163697 PMC7121643

[10] Novel Swine-Origin Influenza A (H1N1) Virus Investigation Team, Dawood FS, Jain S, Finelli L, Shaw MW, Lindstrom S, Garten RJ, Gubareva LV, Xu X, Bridges CB, Uyeki TM 2009 Emergence of a novel swine-origin influenza A (H1N1) virus in humans. N Engl J Med 360:2605–2615. doi:10.1056/NEJMoa0903810. 19423869

